# Is BMI a Valid Indicator of Overweight and Obesity for Adolescents?

**DOI:** 10.3390/ijerph17134815

**Published:** 2020-07-04

**Authors:** Viktoryia Karchynskaya, Jaroslava Kopcakova, Daniel Klein, Aleš Gába, Andrea Madarasova-Geckova, Jitse P. van Dijk, Andrea F. de Winter, Sijmen A. Reijneveld

**Affiliations:** 1 Department of Health Psychology and Research Methodology, Faculty of Medicine, P.J. Safarik University in Kosice, 040 01 Kosice, Slovakia; jaroslava.kopcakova@upjs.sk (J.K.); andrea.geckova@upjs.sk (A.M.-G.); 2 Graduate School Kosice Institute for Society & Health, P.J. Safarik University in Kosice, 040 01 Kosice, Slovakia; j.p.van.dijk@umcg.nl; 3 Department of Community & Occupational Medicine, University Medical Center Groningen, University of Groningen, 9713AV Groningen, The Netherlands; a.f.de.winter@umcg.nl (A.F.d.W.); s.a.reijneveld@umcg.nl (S.A.R.); 4 Institute of Mathematics, Faculty of Natural Sciences, P.J. Safarik University in Kosice, 040 01 Kosice, Slovakia; daniel.klein@upjs.sk; 5 Department of Natural Sciences in Kinanthropology, Faculty of Physical Culture, Palacky University in Olomouc, 771 11 Olomouc, Czech Republic; Ales.Gaba@upol.cz; 6 Olomouc University Social Health Institute, Palacky University in Olomouc, 771 11 Olomouc, Czech Republic

**Keywords:** adolescents, overweight, obesity, body mass index, body fat proportion

## Abstract

*Background*: Overweight and obesity are mostly monitored via the Body Mass Index (BMI), based on self-reported or measured height and weight. Previous studies have shown that BMI as a measure of obesity can introduce important misclassification problems. The aim of this study was to assess the validity of overweight and obesity classification based on self-reported and on measured height and weight versus the proportion of body fat as the criterion. *Methods*: We used data on 782 adolescents (mean age = 13.5, 55.8% boys) from the Health Behaviour in School-Aged Children (HBSC) study conducted in 2018 in Slovakia. We obtained self-reported (height and weight) and objective measures (height, weight) and the proportion of fat (as the criterion measure) measured via bioimpedance body composition analysis (BIA) with an InBody 230 from the adolescents. *Results*: Both measured and self-reported BMI indicated overweight and obesity with relatively low sensitivity (66–82%), but high specificity (90–92%). The superior accuracy of measured BMI in comparison to self-reported BMI was confirmed by the area under the curve (AUC) based on the receiver operating characteristics (ROC) curves (AUC measured/self-reported: 0.94/0.89; *p* < 0.001). The misclassification of overweight and obesity was significantly higher when using self-reported BMI than when using measured BMI. *Conclusion*: Both self-reported and measured BMI as indicators of overweight and obesity underestimate the prevalence of adolescents with overweight and obesity.

## 1. Introduction

Childhood obesity is one of the most serious and alarming of public health challenges today [1,2]. In the past 40 years, the number of children with obesity worldwide has increased 10-fold. Thus, there are now about 124 million children and adolescents in the world with obesity [3]. These estimates are mostly based on weight and height as measures used to determine overweight and obesity. However, these categories have also been defined by the World Health Organization (WHO) [4] in a more biological way, i.e., as an abnormal or excessive fat accumulation that may impair health. Worryingly, childhood obesity is quite likely to continue into adulthood, where it leads to health issues such as diabetes, cardiovascular diseases, and oncological diseases [5]. In association with this, obesity also leads to a very high demand for healthcare and to high societal costs.

To maintain the health and well-being of youth and to reduce the burden of obesity-related diseases in adulthood, early diagnosis and treatment of overweight and obesity in childhood and adolescence is urgently called for. Pediatric screening for overweight and obesity relies mostly on the Body Mass Index (BMI), derived from measured or self-reported height and weight [6]. BMIs based on both types of data have disputable validity, although measured BMI may be relatively better [7,8]. A first problem is that self-reports may be biased compared to measured weight and height [8,9,10]. Previous studies have shown that self-reported weights are significantly lower than measured weights [7,11,12]. Several explanations have been proposed for this. First, the stigma of being overweight or obese may cause people to underestimate their weight due to cognitive dissonance [13,14]. Second, according to the visual normalization theory regarding underestimation of overweight and obesity [15], the reason for this underreporting may be that the number of larger body sizes has increased. This increase in numbers may then lead to an increase in the visual threshold and a recalibration of the body weight range that is perceived as “normal”.

A second problem may be that BMI does not adequately represent overweight and obesity, whether derived from self-reported or measured weight and height [16]. This indicator may work very well for children with a normal proportion of fat and muscles, but may be misleading in children with either a higher proportion of muscles or a higher proportion of fat [16]. This suggests that BMI as a measure of obesity can introduce important misclassification problems [17,18], i.e., resulting in counting children with overweight or obesity as having normal weight and vice versa. When such a misclassification occurs with a certain frequency, it should be identified as important.

Measuring total body fat is considered to be a better measurement, because this type of measurement yields estimates of lean mass or fat mass [16,19,20]. Measured body fat percentage can thus be a criterion for measuring overweight and obesity among adolescents. However, in practice, this is relatively difficult to measure, making it unsuitable for use in routine care [21]. Therefore, overweight and obesity as risk factors for morbidity and mortality should be regularly monitored, but are mostly monitored via BMI based on self-reported or measured height and weight. Evidence is lacking, however, on the combination three different indicators (self-reported BMI, measured BMI, and proportion of fat) of overweight and obesity among adolescents aged 11 to 15 years, at least in Central and Eastern Europe. Accordingly, the aim of this study is to assess the validity of overweight and obesity based on self-reported and on measured height and weight versus using the proportion of fat as the criterion.

## 2. Materials and Methods

### 2.1. Sample and Procedure

We used data on 782 adolescents (mean age = 13.5, 55.8% boys) from the Health Behaviour in School-Aged Children (HBSC) study conducted in 2018 in Slovakia. These constituted a random sample of about 9.3% of all children participating in the HBSC study. We used three-step sampling to obtain a representative sample. In the first step, 140 larger and smaller elementary schools located in rural and urban areas from all regions of Slovakia were asked to participate. These were randomly selected from a list of all eligible schools in Slovakia, obtained from the Slovak Institute of Information and Prognosis for Education. School response rate (RR) was 77.9%; student RR in selected schools was 60.1%, and student RR for body measurements was 81.8%. In the second step, we obtained data from 8405 adolescents from the fifth to ninth grades of elementary schools in Slovakia in the target group of 11 to 15 year olds (mean age = 13.4; 50.9% boys). In the third step, 10% of elementary schools were randomly selected from the total sample of the HBSC study for anthropometric measurements (body height, body weight, and body composition), leading to 888 adolescents being measured. From these, we excluded 106 adolescents during the data-cleaning phase (11.6%) because of missing data on self-reported weight, height or weight, and height of adolescents (i.e., making it impossible to calculate BMI based of self-reported data), or due to other specific errors in the self-report questionnaire (e.g., unspecified gender, age, etc.). This led to a final study sample of 782 adolescents aged 11 to 15 years old.

The study was approved by the Ethics Committee of the Medical Faculty at the P.J. Safarik University in Kosice (16N/2017). Parents were informed about the study via the school administration and could opt out if they disagreed with their child’s participation. Participation in the study was fully voluntary and anonymous with no explicit incentives provided for participation.

### 2.2. Measures

#### 2.2.1. Anthropometric Data and Assessment of Body Fat

Regarding anthropometric data, we measured height, weight, and body fat percentage. We measured body height using the Anthropometer A226 (TRYSTOM Co., Ltd., Olomouc, Czech Republic). We carried out the measurements according to the following measurement protocol [22]: before the measurement, adolescents assumed an active upright position and maintained it throughout the measurement. The position of the head was standardized by asking the respondent to stand straight, without shoes and with the heels together. Adolescents stood with their backs to a vertical wall, with their heels and toes together. The heels, buttocks, and shoulder blades touched the wall, with the head oriented in the so-called Frankfurt horizontal plane.

We measured body weight (kg) and fat percentage (%) via bioimpedance body composition analysis (BIA) with an InBody 230 (Biospace Co., Ltd., Seoul, Korea). The analysis was carried out according to the manufacturer’s instructions [23]. Before the measurements were made, the adolescents were instructed to be dressed in a maximum of a t-shirt and trousers or a skirt. The starting weight was set at −0.5 kg, in order to take into account that we were not weighing the adolescents in underwear. Boys and girls with a proportion of body fat of over 25% and 30%, respectively, were considered to be overweight or obese [18,20].

#### 2.2.2. Self-Reported Data

Self-reported data included information about body height and body weight provided by the adolescents themselves. Body height was obtained via the single-item question from the HBSC questionnaire: “How tall are you with no clothes on?” in cm [1]. We obtained body weight via the single-item question from the HBSC questionnaire: “How much do you weigh with no clothes on?” in kg [1].

Family affluence was measured using the Family Affluence Scale III (FAS-III), which consists of six questions: “Does your family own a car, van, or truck?” (No/Yes, One/Yes, two or more), “Do you have your own bedroom for yourself?” (Yes/No), “How many computers does your family own?” (None/ One/Two/More than two), “How many bathrooms (room with a bath/shower or both) are in your home?” (None/One/Two/More than two), “Does your family have a dishwasher at home?” (Yes/No), “How many times did you and your family travel out of your country for a holiday/vacation last year?” (Not at all/Once/Twice/More than twice). We computed the sum score, which we converted to a ridit score ranging from 0 to 1. We then created tertile categories of low (0 to 0.333), medium (0.334 to 0.666), and high (0.667 to 1) socioeconomic position [24].

#### 2.2.3. Overweight and Obesity

Body Mass Index (BMI) was calculated using the formula BMI = kg/m^2^ from measured and self-reported height and weight, and the WHO BMI z-scores were also calculated [25]. The commonly used 1 standard deviation (SD) cut-off point was then used to indicate adolescents with overweight, and a 2 standard deviation cut-off point was used to indicate adolescents with obesity [25].

With regard to body fat percentage, the cut-off points of 25% for boys and 30% for girls were used to indicate adolescents with overweight or obesity.

### 2.3. Statistical Analyses

First, we described the characteristics of the sample and the prevalence of overweight and obesity as assessed using various indicators. Second, we assessed the validity of self-reported BMI and measured BMI, calculating sensitivity and specificity using the proportion of fat as the criterion measure and the positive and negative predictive value. Sensitivity is the probability that BMI correctly identifies adolescents with overweight and obesity according to the criterion of measured fat percentage, and specificity is the probability that BMI correctly reveals normal weight according to that criterion. We further computed the area under the curve (AUC) based on the receiver operating characteristics (ROC) curves to obtain summary statistical measures of the tests’ diagnostic discrimination abilities for both measured and self-reported BMI, and then computed the 95% confidence interval (CI) for the AUCs. A ROC curve is a measure for the performance of a test compared to the criterion (body fat percentage) at various threshold settings of the test. The area under the ROC curve (AUC) helped to summarize this performance. It summarized the degree to which the test adds to the prediction at the various threshold cut-offs, i.e., the degree to which the test (i.e., BMI) can distinguish between adolescents with overweight or obesity and with normal weight. The positive predictive value (PPV) and negative predictive value (NPV) focused on the predictive power of BMI. The PPV represents the probability that if the BMI classifies an adolescent as having obesity, he or she has obesity according to the criterion. The NPV represents the probability that if the BMI classifies an adolescent as having normal weight, he/she has a normal weight according to the criterion. The optimal BMI z-score cut-off point for measured and self-reported BMI was defined as a BMI z-score value providing the highest possible Youden index [26]. The data were analyzed in IBM SPSS version 23.0 (IBM, New York, United States) and Stata 11 (StataCorp LLC, Texas, United States).

## 3. Results

**Figure 1:**
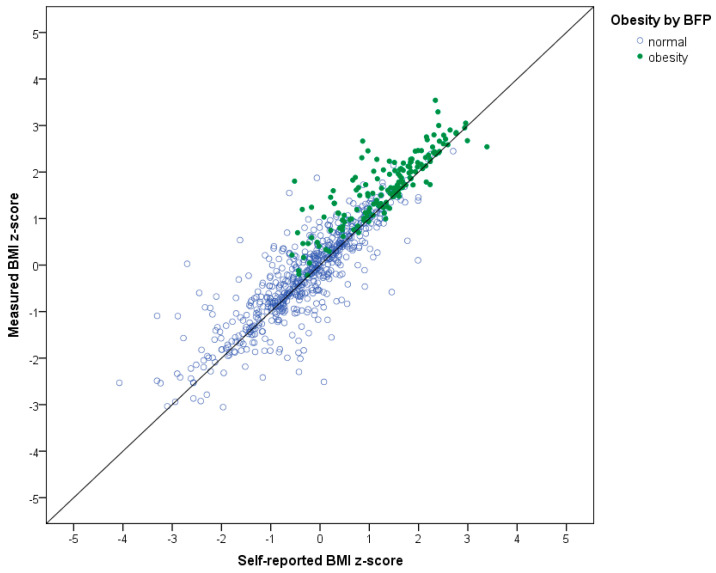
z-Scores of measured and self-reported BMI classified with overweight/obesity based on body fat percentage.

The sample consisted of slightly more boys (56%) than girls, and 15–20% of the sample was overweight or obese according to the three measures: body fat percentage, measured BMI, and self-reported BMI (Table 1). Figure 1 shows the considerable number of misclassifications of adolescents with overweight and obesity by both measured and self-reported BMI.

**Figure 2:**
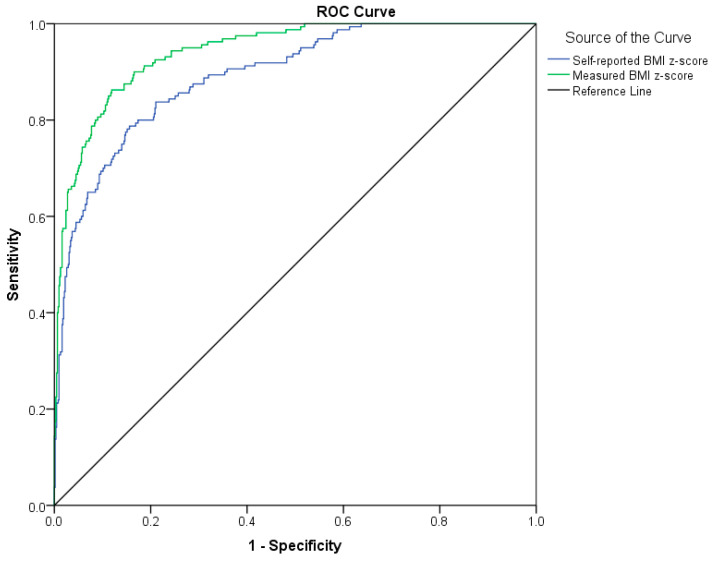
Receiver operating characteristics (ROC) curve for measured BMI and self-reported BMI.

Both measured and self-reported BMI indicated overweight and obesity measured by body fat proportion with relatively high specificity (90–92%), but lower sensitivity (66–82%) (Table 2). Moreover, measured and self-reported BMI significantly differed in sensitivity, but not in specificity. A rather large number of adolescents with overweight and obesity were misclassified as having normal weight (34% by self-reported BMI and 18% by measured BMI); the degree of misclassification was significantly larger for BMI based on self-reported data for height and weight (Table 2). This better accuracy of measured BMI was confirmed by the AUC (Figure 2). If we considered adolescents with a proportion of body fat higher than 25%, or 30%, to suffer from obesity, the indices of validity changed. Measured and self-reported BMI indicated obesity measured by body fat proportion with higher specificity (99%), but lower sensitivity (21–35%) (Table 3). Moreover, measured and self-reported BMI significantly differed in sensitivity, but not in specificity. The better accuracy of measured BMI in comparison to self-reported BMI was confirmed by the area under a ROC curve (AUC measured 0.94 (95% CI 0.92–0.96), AUC self-reported 0.89 (95% CI 0.86–0.92); *p* < 0.001), and this better accuracy of measured BMI was confirmed by the AUC (Figure 2). It was estimated that the best cut-off point for identifying obesity was 0.92 (sensitivity = 86%, specificity = 0.88%, Youden index = 0.74) for measured BMI z-score, and 0.66 (sensitivity = 79%, specificity = 0.84%, Youden index = 0.63) for self-reported BMI z-score.

## 4. Discussion

We assessed the criterion validity of overweight and obesity based on self-reported and measured height and weight versus the proportion of fat in 782 Slovak adolescents from 11 to 15 years old. Both measured and self-reported BMI indicated overweight and obesity with relatively low sensitivity but high specificity. Moreover, misclassification of overweight and obesity was significantly higher when indicated by self-reported BMI in comparison to measured BMI.

We found that self-reported BMI only partially represented obesity and overweight as measured by the criterion (body fat percentage). Previous studies have provided several explanations for errors in self-reported weight and in a person’s overweight status. First, the degree of bias in self-reports has been shown to increase directly with the amount of overweight, and to be much higher for females than for males [27,28]. A second explanation may be the higher tendency of people with lower aerobic fitness to underestimate their weight [29]. These errors may add to the limited validity of overweight or obesity based on self-report.

We also found a discrepancy between measured BMI and the proportion of fat. This discrepancy could be explained in the following ways. A first explanation for such differences is that BMI does not account for the weight of bones, muscles, and fat, as noted by Rothman [16]. A second explanation is that in our analyses, we did not account for pubertal stage. Crocker et al. [30] found a significant interaction between sex and obesity in predicting an adolescent’s pubertal development. This indicates that in adolescence, pubertal stage may be another issue to be considered, as it is closely related to variation in body fat and is not consistent with age in a considerable proportion of adolescents. A third explanation is that predicting body fat levels depends on race, gender, and age, as noted by Mills et al. [31]. We cannot differentiate between these explanations based on our findings, but measured BMI seemed to have an acceptable validity and performed relatively better than self-reported BMI. However, it also sometimes misclassified overweight and obesity, and this was more frequent for some groups.

We found a high specificity for not being overweight/obese for both self-reported and measured BMI. This is in line with the previous study of Javed et al. [32], which showed that BMI had high specificity but low sensitivity for detecting excess adiposity, and fails to identify adolescents with excess body fat percentage. In addition, our study showed that the indicator of sensitivity was not high enough, especially the sensitivity of self-reported BMI. Prentice [33] believes that BMI cut-offs are not specific enough and may lead to the incorrect classification of children with normal weight as obese. However, according to Reilly et al. [34], more attention needs to be paid to the low sensitivity of BMI cut-offs, which shows them to be of limited value in identifying children with obesity. The prevalence of adolescents with overweight and obesity based on self-reported BMI is rather low, although somewhat better by measured BMI. It is important to note that during a similar study on the adult population, the high specificity and low sensitivity of BMI were also established, as more than half of people with obesity were misclassified [35]. This indicates that BMI as an indicator of overweight and of obesity in particular underestimates the real rate of adolescents with overweight and obesity. Both self-reported and measured BMI are good measurements for identifying adolescents with overweight or obesity, and sensitivity is considerably increased with the use of the proposed cut-off points for adolescents with obesity.

The major strengths of this study relate to its large sample of adolescents and the three different types of measurements used in the study of overweight and obesity among adolescents.

Some limitations should also be mentioned. The main limitation of this study relates to the cut-offs of body fat percentage that were used to identify overweight and obesity among adolescents. In this case, gender was used to separate in the cut-offs of body fat percentage in adolescents. However, the generally accepted cut-offs of adolescent body fat percentage at different stages of maturation have not yet been established [36,37]. According to Pinto et al. [38], the prevalence of overweight and abdominal obesity showed an increase in the final stages of sexual maturation for both sexes, when indicators of BMI and waist circumference were evaluated. However, the effects of obesity on early puberty in boys are more contentious, and require the development of robust biomarkers [37]. In addition, a potential limitation of our study is the use of bioelectrical impedance analysis as a criterion to assess body fat. We did so because this marker has been widely used in studies to examine the diagnostic performance of BMI to identify obesity in the pediatric population [32], and our choice has been supported by different studies [16,20,39]. However, the use of other methods for adiposity assessment (for example, the fat mass index) could lead to somewhat different findings.

We found that both self-reported and measured BMI led to an underestimation of the prevalence of overweight and obesity in epidemiological studies. This may contribute to misclassification in pediatric and educational centers’ practice. It may imply that estimates of the prevalence of overweight and obesity should be adjusted for this, with degrees depending on possible predictors of this bias (e.g., gender, age, socioeconomic status, weight status). Even though BMI based on self-reported data of weight and height is a quick, cheap, and easy-to-implement measure to identify overweight and obesity, there are several reasons to use it with caution. First, it might considerably underestimate the real prevalence of overweight and obesity in the population and might thus inform health promotion interventions incorrectly if it not adjusted. Second, it might bias expert and lay awareness about the size of this public health problem, leading to limited support for policy actions. Third, it might influence the beliefs of adolescents about their body composition. This may negatively affect their health literacy and may decrease their willingness to improve their health behaviors. All in all, this may increase the burden of chronic diseases and comorbidities due to obesity, such as impaired glucose tolerance, type 2 diabetes, hypertension, and hepatic steatosis [5,6], and thus highly negatively affect health at both the individual and societal level. Pediatricians should thus combine self-reports of weight and height with measurements of these characteristics or, preferably, use other measures like body fat proportion or waist and hip proportion. Moreover, both in practice and research, we should take into account the puberty stage of adolescents. These new findings based on adolescents from Central and Eastern Europe definitely require confirmation using other criteria, such as fat mass index, and in other settings to be able to assess the effects of contextual factors.

## 5. Conclusions

Self-reported BMI as an indicator of overweight and obesity significantly underestimates the prevalence of adolescents with overweight and obesity. Measured BMI will detect adolescents with overweight and obesity with greater accuracy, but even using measured height and weight may lead to underestimation of overweight/obesity and bias.

## Figures and Tables

**Table 1:** Characteristics of the sample (N = 782, 11–15-year-old Slovak school-aged children, data collected in 2018).

Characteristics	N (%)
Gender	
Boys	436 (55.8)
Girls	346 (44.2)
Age	
11 years	121 (15.5)
12 years	173 (22.1)
13 years	188 (24.0)
14 years	185 (23.7)
15 years	115 (14.7)
Family affluence	
Low	152 (24.9)
Middle	183 (30.0)
High	275 (45.1)
Number and percentage of adolescents with overweight based on:	
BMI from measured height and weight	136 (17.4)
BMI from self-reported height and weight	121 (15.5)
Number and percentage of adolescents with obesity based on:	
BMI from measured height and weight	60 (7.7)
BMI from self-reported height and weight	37 (4.7)
Body fat percentage (adolescents with overweight or obesity)	160 (20.5)

Note: BMI—body mass index.

**Table 2:** The validity of overweight and obesity classification by measured BMI and self-reported BMI measured by several diagnostic indices.

Diagnostic Indices	Measured BMI	Self-Reported BMI	*p*-Value of Difference *
Sensitivity	82% (CI 75–87%)	66% (CI 58–73%)	<0.001
Specificity	90% (CI 87–92%)	92% (CI 89–94%)	0.058
PPV	67% (CI 60–73%)	67% (CI 58–74%)	
NPV	95% (CI 93–97%)	91% (CI 87–93%)	

Note: BMI—Body Mass Index; PPV—positive predictive value; NPV—negative predictive value; CI—95% confidence interval; * based on McNemar’s test.

**Table 3:** The validity of obesity by measured BMI and self-reported BMI measured by several diagnostic indices.

Diagnostic Indices	Measured BMI	Self-reported BMI	*p*-Value of Difference *
Sensitivity	35% (CI 28–43%)	21% (CI 15–28%)	<0.001
Specificity	99% (CI 98–100%)	99% (CI 98–100%)	≈1
PPV	93% (CI 83–98%)	92% (CI 78–97%)	
NPV	86% (CI 83–88%)	83% (CI 82–84%)	

Note: BMI—Body Mass Index; PPV—Positive predictive value; NPV—Negative predictive value; CI—95% Confidence Interval; * Based on McNemar’s test.
